# Converting networks to predictive logic models from perturbation signalling data with CellNOpt

**DOI:** 10.1093/bioinformatics/btaa561

**Published:** 2020-06-09

**Authors:** Enio Gjerga, Panuwat Trairatphisan, Attila Gabor, Hermann Koch, Celine Chevalier, Franceco Ceccarelli, Aurelien Dugourd, Alexander Mitsos, Julio Saez-Rodriguez

**Affiliations:** Faculty of Medicine, Heidelberg University, Heidelberg University Hospital, Institute for Computational Biomedicine, BioQuant 69120 Heidelberg, Germany; Faculty of Medicine, Joint Research Centre for Computational Biomedicine (JRC-COMBINE); Faculty of Medicine, Heidelberg University, Heidelberg University Hospital, Institute for Computational Biomedicine, BioQuant 69120 Heidelberg, Germany; Faculty of Medicine, Heidelberg University, Heidelberg University Hospital, Institute for Computational Biomedicine, BioQuant 69120 Heidelberg, Germany; Faculty of Medicine, Joint Research Centre for Computational Biomedicine (JRC-COMBINE); Aachener Verfahrenstechnik, Process Systems Engineering, RWTH Aachen University, Aachen, Germany; Faculty of Medicine, Joint Research Centre for Computational Biomedicine (JRC-COMBINE); University Paris-Saclay, Espace Technologique Bat. Discovery,91190 Saint-Aubin, France; Faculty of Medicine, Joint Research Centre for Computational Biomedicine (JRC-COMBINE); Computer Laboratory, University of Cambridge, Cambridge CB2 1TN, UK; Faculty of Medicine, Heidelberg University, Heidelberg University Hospital, Institute for Computational Biomedicine, BioQuant 69120 Heidelberg, Germany; Faculty of Medicine, Joint Research Centre for Computational Biomedicine (JRC-COMBINE); Aachener Verfahrenstechnik, Process Systems Engineering, RWTH Aachen University, Aachen, Germany; Faculty of Medicine, Heidelberg University, Heidelberg University Hospital, Institute for Computational Biomedicine, BioQuant 69120 Heidelberg, Germany; Faculty of Medicine, Joint Research Centre for Computational Biomedicine (JRC-COMBINE)

## Abstract

**Summary:**

The molecular changes induced by perturbations such as drugs and ligands are highly informative of the intracellular wiring. Our capacity to generate large datasets is increasing steadily. A useful way to extract mechanistic insight from the data is by integrating them with a prior knowledge network of signalling to obtain dynamic models. CellNOpt is a collection of Bioconductor R packages for building logic models from perturbation data and prior knowledge of signalling networks. We have recently developed new components and refined the existing ones to keep up with the computational demand of increasingly large datasets, including (i) an efficient integer linear programming, (ii) a probabilistic logic implementation for semi-quantitative datasets, (iii) the integration of a stochastic Boolean simulator, (iv) a tool to identify missing links, (v) systematic post-hoc analyses and (vi) an R-Shiny tool to run CellNOpt interactively.

**Availability and implementation:**

R-package(s): https://github.com/saezlab/cellnopt.

**Supplementary information:**

Supplementary data are available at *Bioinformatics* online.

## 1 Introduction

**Fig. 1.**
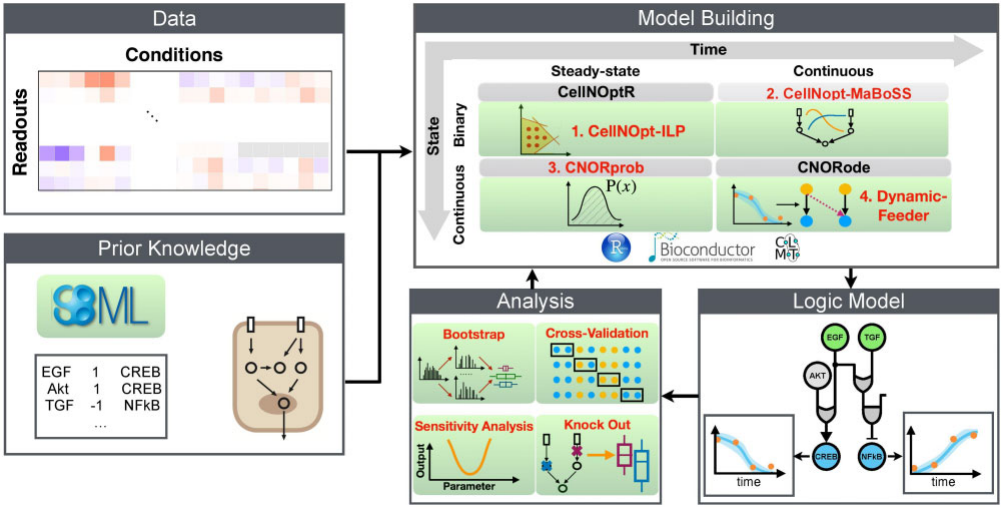
CellNOpt pipeline with packages and features. Perturbation data are combined with prior knowledge of signalling interactions. Different modules can be used for model building: CellNOptR (Boolean logic models), CellNOpt-MaBoSS (stochastic simulations), CNORprob (probabilistic formalism), CNORode (logic-based ODEs). New packages and features are highlighted with green background and red text. (Color version of this figure is available at *Bioinformatics* online.)

Logic networks are among the conceptually simplest modelling frameworks. They capture the mechanistic relationship between molecular entities by logic gates. Due to their simplicity, they are highly scalable and widely applied. We have previously introduced CellNetOptimiser (CellNOpt), a framework to build predictive logic models of signalling pathways by training a prior knowledge network (PKN) to biochemical data obtained from perturbation experiments (Terfve *et al.*, 2012). The CellNOpt R packages feature different formalisms ranging from Boolean logic to logic-based ordinary differential equations(Fig. 1). The value of CellNOpt has been recently demonstrated on several applications(Supplementary Table S1).

Over the past years, we have continuously added new features and upgrades to the packages to enhance the functionality and computational efficiency of CellNOpt (Fig. 1). The current toolkit covers a unique set of features (see Supplementary Table S2). Furthermore, for alternative analyses, CellNOpt supports importing from and exporting to other tools via simple interaction files and the SBMLQual (Chaouiya *et al.*, 2013) formats.

## 2 Summary of new features

We enumerate here the new features that enrich the CellNOpt framework.

### 2.1 CellNOpt-ILP

For the training of Boolean logic networks, we implemented an integer linear programming (ILP) formulation based on (Mitsos *et al.*, 2009) and the IBM CPLEX optimizer. This allows us to optimize large-scale networks orders of magnitude faster than the built-in genetic algorithm. Furthermore, the method can generate pools of (near) optimal solutions, which helps identify uncertainty in the inferred network structure. We illustrate the functionality of CellNOpt-ILP on several case studies (Supplementary Text S1).

### 2.2 CellNOpt-MaBoSS

MaBoSS (Stoll *et al.*, 2012) performs asynchronous stochastic simulations of logic Boolean models. It was integrated with CellNOpt to optimize Boolean networks where nodes are represented by their state probability with respect to time (Supplementary Text S2).

### 2.3 CNORprob

To optimize logic networks with semi-quantitative states (between 0 and 1) at quasi-steady-state, we offer the CNORprob package. CNORprob is an R-implementation of the Matlab-based toolbox FALCON for probabilistic Boolean logic (De Landtsheer *et al.*, 2017) (Supplementary Text S5).

### 2.4 Dynamic-Feeder

Dynamic-Feeder is a method to identify missing links in the PKN and provides candidates to fill knowledge gaps (Supplementary Fig. S3). It combines data-driven network inference with a protein–protein interaction network to find missing elements. For the latter, we primarily use OmniPath (Turei *et al.*, 2016). Dynamic-Feeder generalizes our previous tool CNORfeeder (Eduati *et al.*, 2012) to time-course data with a logic ordinary differential equations formalism (Supplementary Text S4).

### 2.5 Post-hoc analysis

After optimized logic models are obtained, the predictive power of the models can be assessed by cross-validation and bootstrapping. Further, the package offers parameter sensitivity analysis, and estimation of node and edge essentiality by removing them (knockout). This allows us to observe how sensitive specific proteins/interactions to perturbations are (Supplementary Text S5).

### 2.6 Shiny application

To build and train models with CellNOpt, CNORprob and CNORode without coding, we offer an interactive R-Shiny application (Supplementary Text S6).

In summary, the new CellNOpt features expand the options for logic modelling, in particular to analyze large datasets.

## Supplementary Material

btaa561_supplementary_data

## References

[btaa561-B1] Chaouiya C. et al (2013) SBML qualitative models: a model representation format and infrastructure to foster interactions between qualitative modelling formalisms and tools. BMC Sys. Biol., 7, 135. 10.1186/1752-0509-7-135 PMC3892043 24321545

[btaa561-B2] Eduati F. et al (2012) Integrating literature-constrained and data-driven inference of signalling networks. Bioinformatics, 28, 2311–2317. 22734019 10.1093/bioinformatics/bts363 PMC3436796

[btaa561-B4] Mitsos A. et al (2009) Identifying drug effects via pathway alterations using an integer linear programming optimization formulation on phosphoproteomic data. PLoS Comput. Biol., 5, e1000591. 19997482 10.1371/journal.pcbi.1000591 PMC2776985

[btaa561-B5] Stoll G. et al (2012) Continuous time Boolean modeling for biological signaling: application of Gillespie algorithm. BMC Syst. Biol., 6, 116. 22932419 10.1186/1752-0509-6-116 PMC3517402

[btaa561-B6] Terfve C. et al (2012) CellNOptR: a flexible toolkit to train protein signaling networks to data using multiple logic formalisms. BMC Syst. Biol., 6, 133. 23079107 10.1186/1752-0509-6-133 PMC3605281

[btaa561-B7] Turei D. et al (2016) OmniPath: guidelines and gateway for literature-curated signaling pathway resources. Nat. Methods, 13, 966–967. 27898060 10.1038/nmeth.4077

